# Visually-guided gait training in paretic patients during the first rehabilitation phase: study protocol for a randomized controlled trial

**DOI:** 10.1186/s13063-016-1630-8

**Published:** 2016-10-27

**Authors:** Cathia Rossano, Philippe Terrier

**Affiliations:** 1 IRR, Institute for Research in Rehabilitation, Sion, Switzerland; 2 Clinique romande de réadaptation SUVACare, Av. Gd-Champsec 90, 1951 Sion, Switzerland

**Keywords:** Stroke, Traumatic brain injury, Spinal cord injury, Rehabilitation, Augmented reality, Randomized controlled trial

## Abstract

**Background:**

After a lesion to the central nervous system, many patients suffer from reduced walking capability. In the first rehabilitation phase, repeated walking exercises facilitate muscular strength and stimulate brain plasticity and motor relearning. However, marked limping, an unsteady gait, and poor management of obstacle clearance may persist, which increases a patient’s risk of falling. Gait training with augmented reality has been recommended to improve gait coordination. The objective of this study is to test whether a gait rehabilitation program using augmented reality is superior to a conventional treadmill training program of equivalent intensity.

**Methods/design:**

The GASPAR trial (Gait Adaptation for Stroke Patients with Augmented Reality) is a pragmatic, parallel-arm, single-center, nonblind, superiority randomized control trial in neurorehabilitation. The setting is a rehabilitation clinic in Switzerland. The planned number of participants is 70–100. The intervention uses instrumented treadmills equipped with projectors that display shapes on the walking surface. The principle is that patients must adapt their gait to the image that unfolds in front of them. Specific exercises for gait symmetry, coordination enhancement, and gait agility are provided. The program includes twenty 30-min sessions spanning 4 weeks. The comparator group receives standard treadmill training of a similar frequency and intensity. The main outcome to be measured in the trial is walking speed, which is assessed with the 2-min Walk Test. Moreover, gait parameters are recorded during the gait training sessions. Other outcomes are balance control (Berg Balance Scale) and the fear of falling (Falls Efficacy Scale). The statistical analyses will compare the baseline assessment for each participant (before the intervention) with a post-intervention assessment (taken a few days after the end of the program). Furthermore, a follow-up assessment will take place 3 months after discharge.

**Discussion:**

The study results will provide new knowledge about recovery in neurological patients and will contribute to the design of better rehabilitation programs to accompany this process. The findings will also help health care funders to decide whether treadmills equipped with augmented reality capabilities are a worthwhile investment.

**Trial registration:**

ClinicalTrials.gov ID: NCT02808078, registered on 16 June 2016.

## Background

In high-income countries, strokes are the third leading cause of death after cardiovascular diseases and cancers. In Switzerland, about 16,000 people suffer a stroke each year [1]. However, over the last few decades, the death rate following strokes has continuously declined in developed countries because of improvements in primary care. The corollary to this trend is an increased burden on rehabilitation units because of stroke survivors with severe disabilities [2]. The major long-term disability that hinders stroke survivors in recovering a normal life is partial paralysis affecting the extremities. Six months after a stroke, 50 % of stroke survivors still suffer some degree of hemiparesis and 30 % are unable to walk without assistance [3].

In addition to strokes, other circumstances that damage neural tissue can induce similar disabilities. Traumatic brain injuries (TBI), most commonly induced by falls or road traffic accidents, are also a significant issue in Switzerland, with about 850 severe cases per year [4]. Patients with traumatic spinal cord injuries (SCI), representing about 150 cases per year, can be added to this number [5]. Roughly 50 % of these patients suffer from incomplete injuries and thus exhibit residual locomotor capacities [6].

During the months following an injury to the central nervous system, patients partially recover motor and sensory patterns that were in place before the injury. In stroke patients, the maximum degree of recovery typically occurs within the first 3 months, then it progresses at a slower rate until 6 months have passed [7]. This recovery process is in part driven by brain plasticity. In short, redundant connectivity exists in the brain, and new functional circuits can form via remapping between cortical regions [8, 9]. It has been shown that providing an enriched environment stimulates plasticity and facilitates recovery [9]. Although further studies are needed to better understand brain plasticity in humans [10], neurorehabilitation is largely based on the premise that a set of well-chosen exercises can stimulate neural plasticity and enhance the recovery process.

The following are general guidelines for early neurorehabilitation [11]: (1) care provided by a multidisciplinary team, working together in close cooperation to improve a patient’s independence, (2) task-specific training, in which specific functional tasks are practiced repeatedly, and (3) high-intensity practice. This approach applies to both stroke survivors and TBI patients [12]. It is likely that, in the case of stroke survivors specifically, strength training, overground walking training, and speed-dependent treadmill training can improve aspects of gait [13–16]. To be effective, walking training should occur with an average frequency of 3–5 times weekly, in sessions lasting from 20 to 60 min [15]. Concerning patients with incomplete SCI, strong evidence also exists that repeated walking exercises enhance locomotor recovery [6]. Specifically, task-oriented physiotherapy and manual/robotic treadmill training with bodyweight support have been recommended to stimulate brain plasticity [17].

A favorite target for neurorehabilitation is dynamic and static balance. Patients with gait disorders of neurological etiologies are prone to frequent falls [18–20]. Such falls are induced by altered gait coordination which reduces the patient’s adaptability to environmental demands. A major cause of coordination deficit is asymmetry in the propulsive forces between limbs. Altered proprioception [21], gait ataxia, and vestibular disorders [22] may also induce coordination issues. Balance control can also be compromised because of poor central integration of sensory inputs [23]. Although classical interventions have been shown to improve walking speed [16], asymmetric gait patterns and poor coordination may persist [24]. Different approaches have been proposed to improve gait coordination in these patients, such as task-specific walking practice, ankle-foot orthoses, and functional electrical stimulation [25]. Meta-analyses have shown that the best method for training gait coordination in stroke patients is auditory cueing [13, 25]. In this therapy, patients must synchronize their steps with rhythmic auditory stimuli given at a pace that corrects temporal asymmetries of the gait pattern. A recent systematic review has shown that the addition of 30 min of cueing cadence to gait training, four times a week for 4 weeks, is likely to improve the walking ability of stroke patients [26].

In the last decade, the use of visual cues in interventions aimed at improving gait coordination has been proposed. In a manner similar to auditory cueing, the intent of these visual cues is to encourage patients to walk with a more symmetrical gait pattern. The use of augmented reality (AR), i.e., the projection of shapes over the walking surface of treadmills, can also serve to exercise gait agility. It has been proposed that gait adaptability can be increased through a specific program in which visual cues are projected to trigger step adjustments (e.g., target stepping, obstacle avoidance) [27]. Preliminary results in a limited number of chronic stroke patients have shown that visually-guided gait training improved walking speed, balance, and the level of physical activity [27]. The underlying neurophysiological mechanisms for such improvement may be that this type of program allows for better exploitation of alternate sensory modalities to provide feedback regarding the ongoing movement. In addition, it cannot be excluded that stimulating the brain with an enriched visual environment related to the trained task stimulates neural plasticity. Finally, offering a variety of gait exercises may increase a patient’s motivation. Despite these promising findings, the lack of large clinical trials to date prevents us from drawing definitive conclusions about the usefulness of visually-guided stepping for gait rehabilitation.

### Trial objectives

The main goal of the GASPAR (Gait Adaptation for Stroke Patients with Augmented Reality) trial is to assess the efficacy of a treadmill training program that uses visually-guided stepping (AR) during the first phase of rehabilitation for patients with neurological gait disorders following a stroke. In addition, by the use of a pragmatic design, we seek to increase the generalizability and applicability of the study’s potential findings [28]. Consequently, in order to reflect the typical population in a neurorehabilitation center, we include patients with gait disorders of different etiologies; i.e., in addition to stroke patients, the study includes SCI and TBI patients who need gait rehabilitation. The AR program aims at three therapeutic goals: (1) to improve walking speed, which is important to ensure the independence of the patients in their daily lives, (2) to correct gait asymmetry, which may lead to their recovery of a more functional and physiological gait, thus improving gait coordination and reducing their risk of falls, and (3) to exercise gait agility, which may enhance patients’ adaptability to environmental changes while walking and hence again reduce fall risks. We also focus on assessment of the efficiency of the AR intervention. Specifically, we seek to document the patients’ adherence to, and compliance with, the program and to assess their tolerance for AR walking.

## Methods/design

### Trial design

**Fig. 1:**
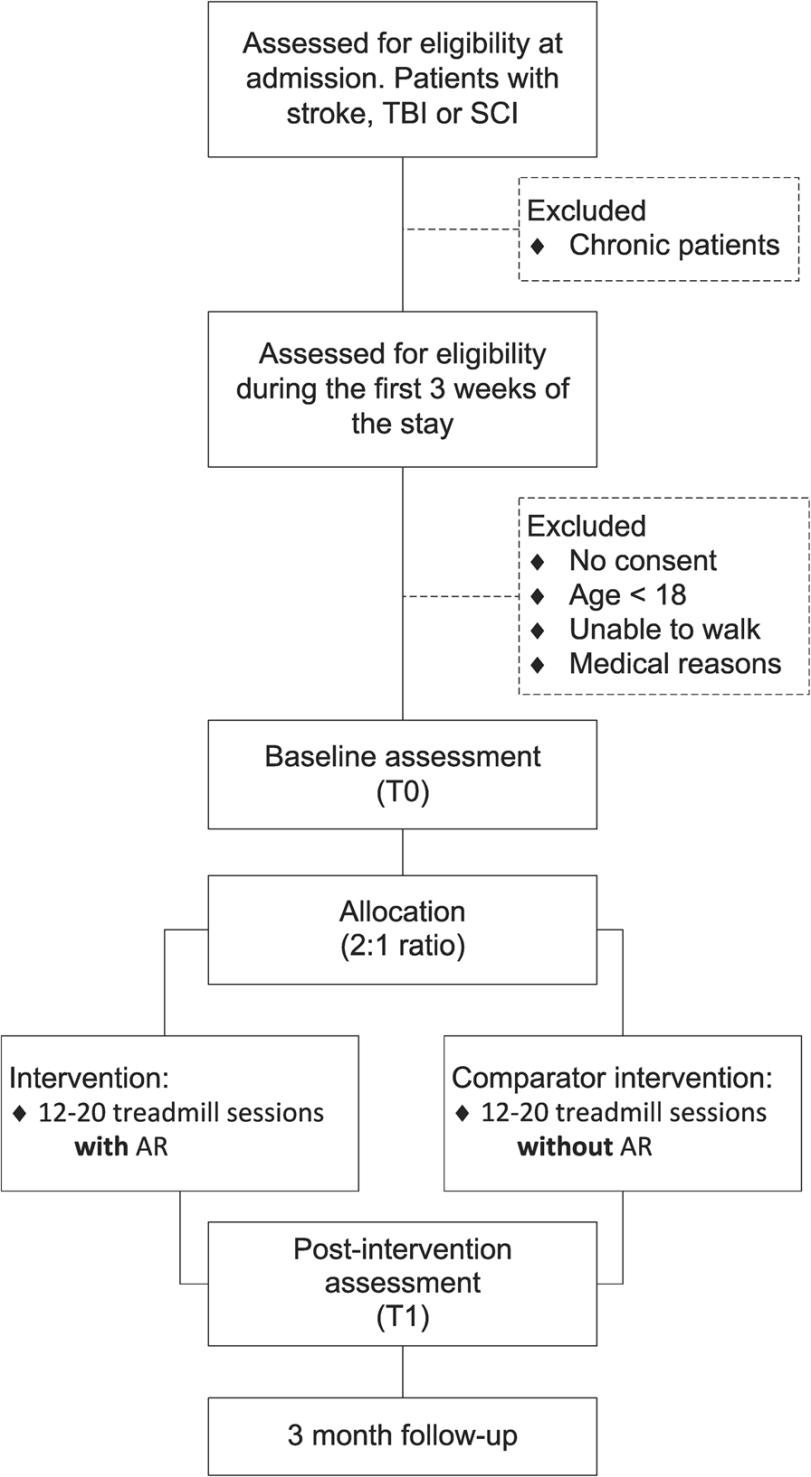
Flowchart of the Gait Adaptation for Stroke Patients with Augmented Reality (GASPAR) trial

The GASPAR trial is a pragmatic, parallel-arm, single-center, nonblind, superiority randomized control trial in neurorehabilitation. The main objective is to test whether a 4-week gait rehabilitation program that uses AR is superior to a conventional treadmill training program of equivalent intensity. The participants’ flow is shown in Fig. 1, and the Standard Protocol Items: Recommendations for Interventional Trials (SPIRIT) schedule [29] is given in Table 1. Baseline assessments (T0) are taken prior to allocation, which consists of blocking randomization (2:1 ratio) with stratification according to disease etiology. Post-intervention assessments of the two groups (T1) serve to compare the short-term efficacy of the different interventions. Three months after discharge, follow-up assessments (T2) take place to detect potential long-term effects.

**Table 1:** Schedule of enrollment, interventions, and assessments according to the SPIRIT guideline

Study periods	Screening	Baseline assessment (T0)	Treatment, intervention period				Post-intervention assessment (T1)	Follow-up (T2)
Time (week)	−3 to −1	0	1	2	3	4	5	15 w after discharge
Patient information and informed consent	x							
Demographics	x							
Medical history	x							
Inclusion/exclusion criteria	x							
Allocation (after T0)		x						
Gait training (Intervention)			x	x	x	x		
Walking speed (2-min Walk Test)		x					x	
Balance (BBS)		x					x	
Gait parameters			x	x	x	x		
Compliance and motivation (questionnaire)				x		x		
Fear of falling (FES)							x	x
Falls Diary								x
Quality of life SF-36								x
Concomitant therapy (usual care for inpatients)	x	x	x	x	x	x	x	
Adverse events		x	x	x	x	x	x	

*BBS* Berg Balance Scale, *FES* Falls Efficacy Scale, *SF-36* Short Form 36-item questionnaire, *w* week

### Setting

The study takes place in the Clinique Romande de Réadaptation (CRR, Sion, Switzerland). This clinic offers 145 beds and is one of the main rehabilitation centers in western Switzerland (with its 2.1 million inhabitants). Because of its affiliation with the accident insurance company SUVA, the CRR is a common referral center for traumatic injuries, especially for SCIs. The overall objective of the CRR’s therapeutic program is to take care of patients through a multidisciplinary approach to improve their quality of life, functional status, and chance of returning to work. The number of hospitalized patients at the CRR is about 1200 per year, with an average stay of 40 days. Recruitment for this study began in June 2016. A 2-year framework is planned for the study, with possible adjustments according to the results of the interim analyses (see below).

### Participants

Study participants will be recruited from among the inpatients admitted for neurorehabilitation at the CRR. Selection of the inclusion criteria was guided by the rationale of reflecting the prevailing choice in neurorehabilitation clinics of specific therapies that are tailored to patients’ needs. A summary of the inclusion/exclusion criteria is shown in Table 2. Potential candidates will be monitored during their first 3 weeks after admission (Fig. 1). Motor functions will be assessed by a skilled neurologist, who will evaluate the need for gait rehabilitation. If and when the candidate is able to walk during a 2-min test, a final decision for inclusion will be made. Those candidates who do not exhibit sufficient walking capacities after 3 weeks will be excluded.

**Table 2:** Inclusion and exclusion criteria

Inclusion criteria	Exclusion criteria
Patient with stroke, traumatic brain injury, or spinal cord injury in acute to subacute phase	Age <18 years
First stage of rehabilitation (<40 days after incident)	Clinically significant concomitant disease states (e.g., cardiovascular disease) that may threaten the patient’s health in case of sustained exercise
Need for gait rehabilitation because of at least one of the following conditions: • Paresis of the lower extremities • Severe balance disorders • No walking at admission	Concomitant gait disorders induced by acute to subacute musculoskeletal injuries (e.g., fracture of the lower extremities)
Ability to walk for 2 min without aid or with minimal aid, namely: • With the help of one person, or: • With walking aid (cane, walker)	Severe preexistent gait disorders that deeply affect walking abilities and gait pattern, either because of musculoskeletal (e.g., severe osteoarthritis) or neurological (e.g., Parkinson’s disease) etiologies
Informed consent as documented by signature	Severe noncorrected visual impairment.
	Inability to follow the procedures of the study, e.g., because of language problems, psychological disorders, dementia, etc.

### Assignment of interventions and blinding

Assignment is effected by a block randomization procedure with varying block size, implemented by independent researchers not affiliated with the GASPAR study. The STATA package RALLOC [30] is used to generate the sequences. The ratio between the intervention and the control group is 2:1; this facilitates recruitment [31] and provides a broader range of information and experience about the AR program. In addition, stratification is made according to the type of injury: stroke patients, TBI patients, and SCI patients. This procedure ensures the comparator and treatment groups to be populated by patients with comparable disease etiologies and thus facilitates interpretation of the results. Sequentially numbered, opaque, sealed envelopes are used to conceal the allocation. Informed consent is obtained by the principal investigator or his/her delegate. Inclusion is confirmed by the referring neurologist. After baseline assessments are taken (T0), the sealed envelope is opened to allocate the participant.

Given the nature of the intervention and its clinical setting, masking is not possible. However, the use of an objective measure as the primary outcome minimizes the risk of bias.

### Medical devices

The intervention uses two different treadmills:

1. The FDM-T treadmill (Zebris Medical, Isny im Allgäu, Germany): this treadmill is equipped with a grid of 10,000 capacitive sensors aimed at analyzing foot pressure (200-Hz sampling rate). The walking surface is 170 × 65 cm. Handrails and a harness ensure optimal safety even for frail individuals. A wide-angle projector (beamer) situated in front of the belt projects stepping targets for the purpose of visual gait training. The Zebris treadmill has been successfully used for gait rehabilitation in patients with Parkinson’s disease [32]
2. The C-Mill treadmill: this treadmill (MotekForce Link, Doorn, The Netherlands) is equipped with an embedded 70 × 300 cm vertical force platform. The long walking surface allows users to exercise gait agility in varying positions along the belt. The treadmill records the vertical force and the position of the center of pressure at a sampling rate of 500 Hz. A projection system displays visual objects on the walking area from the right side of the treadmill. Three recent studies have highlighted the potential of the C-Mill treadmill for stroke rehabilitation [27, 33, 34]

### Intervention

The intervention consists of 20 gait training sessions over 4 weeks. Each training session lasts 30 min. The first 10 sessions (2 weeks) use the Zebris treadmill for basic training of walking speed, step length, and gait symmetry. In the last 2 weeks (10 sessions), the C-Mill treadmill is used for training that is more oriented toward gait agility:

1. Zebris sessions: the gait training performed on the Zebris treadmill aims at improving walking speed, step length, and gait symmetry. The 30-min session begins with a 1-min warmup used to record gait parameters. Then, 5 × 5-min walking intervals are proposed to the participant, punctuated by breaks of 30 s to 1 min. In the first interval, the patient must simply synchronize his/her steps with visual targets (“stepping stones”) based on the gait parameters recorded during the warmup. Then, two types of training are proposed according to the patient’s needs: (1) symmetry training, where the stepping stones are progressively adapted toward a more symmetrical pattern, and (2) step-length training, in which the stepping stones are progressively adapted to present longer steps to the participant. Finally, a 1-min walk without stepping stones is performed at the end of the session to evaluate the immediate effect of the session on the patient’s gait parameters
2. C-Mill sessions: C-Mill sessions aim at training gait agility, that is, speed adaptation and obstacle avoidance. Similar to the Zebris session, the 30-min C-Mill session is divided into 5 × 5-min walking intervals, preceded by a 1-min warmup and followed by a 1-min final assessment. The first interval is dedicated to symmetry or step-length training; the stepping stones are adjusted to provide asymmetry correction or longer steps. In the second interval, the participant must continuously adjust his/her steps to random variations in the position of the stepping stones (varying step length). During the third interval, the patient follows a target area that moves back and forth over the entire length of the treadmill, requiring coordinated speeding up and slowing down. The fourth interval is similar, but with increased difficulty (higher acceleration). Finally, training that includes obstacle avoidance is proposed: unilateral and bilateral obstacles are projected in front of the participant, who must step over them. The speed at which the intervals are performed is kept constant throughout one session, but is progressively increased from one session to another.

The intervention is tailored to the needs and capacity of each participant. For instance, fatigue might make it impossible for a patient to walk for the targeted 25 min, or a frail patient might not make sufficient progress to use the C-Mill after 2 weeks on the Zebris treadmill. Conversely, higher-functioning patients might benefit from C-Mill treatment earlier. The number of intervals performed in each session and the exact training program are documented and taken into account in the statistical analysis.

### Comparator

The choice of intervention for the comparator group reflects a balance between the ethical obligation to provide efficient therapy to all participants and the requirement of standardization to specifically assess the efficacy of AR gait training. Therefore, we exercise participants in the comparator group with the same treadmills and with comparable frequency and intensity to the participants in the intervention group, but without the AR. The walking exercises consist of a progressive speed increase within and between the sessions.

### Concomitant treatments

The interventions are fully integrated into the CRR’s therapeutic program, which includes individual and group therapies (exercises, joint mobilization, speech rehabilitation, etc.) every weekday for a mean time of 2.5 h. The participants in the study follow the usual schedule of care, except that some of the routine physiotherapy sessions are replaced by the study interventions.

### Study outcomes

The selection of the outcomes was guided by three criteria: (1) importance for the patient, (2) ease of implementation in a clinical context, and (3) validity to assess the recovery of locomotor function and gait coordination. A summary of the selected outcomes and their specific aims is presented in Table 3. The principal outcome is walking speed, which is a widely used index in research and in rehabilitation clinics [16]. Furthermore, speed is a good predictor for a patient’s independence during daily life activities [7, 15]. As additional indexes of walking ability, we collect gait data during every training session, taking advantage of the embedded sensors in the treadmills. Typical spatiotemporal parameters are measured: step length, step width, step time, stance phase duration (percentage of gait cycle), and swing phase duration (percentage of gait cycle). Moreover, spatial and temporal left/right asymmetries and stride-to-stride variability are assessed. Recording gait parameters on a daily basis will allow for detailed analysis of the participants’ evolution during the intervention. Other outcomes are related to balance and fall risk, such as the Berg Balance Scale (BBS), a falls efficacy questionnaire (FES), and a Falls Diary after discharge. The assessments (T0, T1) are performed by skilled therapists as part of usual care. Finally, the patients’ subjective perceptions of the intervention and their quality of life at home 3–4 months after discharge are also investigated. Data management follows the clinical practices recommended by the ISO:14155 standard.

**Table 3:** Trial outcomes

Outcomes	Assessments	Objectives
Walking speed	2-min Walk Test [36]	To estimate the overall effect of intervention on walking abilities
Gait parameters	Spatiotemporal parameters (including symmetry) measured by the instrumented treadmills	To measure participant’s gait in each training session for analyzing longitudinally the evolution of walking abilities
Balance	Berg Balance Scale (BBS) [37]	To estimate the effect of intervention on static balance
Fear of falling	Questionnaire: adapted Falls Efficacy Scale (FES) [38]	To assess the participant’s confidence in performing daily living activities that require balance control
Falls	Falls Diary completed by the participant over the 3–4 months following discharge	To measure effective fall rate at home
Perception of the intervention	Ad-hoc questionnaire (tailor-made survey)	To evaluate the patient’s subjective perception of the intervention
Quality of life	Questionnaire: Short Form 36-item (SF-36) questionnaire [39]	To obtain an overall survey of wellbeing and quality of life

### Sample size

An interim analysis based on 30 participants with complete data will be conducted to perform a power analysis and to compute the final sample size. The current estimation (as of June 2016) is that a total of 70 to 100 participants will be recruited. The reasons for such an approach are as follows: the hospitalization statistics for the years 2014 and 2015 show that about 300 patients per year were admitted for neurorehabilitation at the CRR, of which 70 were acute to subacute patients. Retrospective analyses of medical files over recent years indicate that 35–50 patients per year would have been eligible for the study: 20–25 stroke patients, 10–15 TBI patients, and 5–10 SCI patients. As a result, approximately 70–100 eligible candidates are expected over the 2-year framework of the study. The uncertainty of this number results from the substantial year-to-year variability seen in patients’ profiles. A preliminary power analysis is not possible for two reasons: (1) the data currently available in the literature about walking speed in acute to subacute TBI and SCI patients are sparse and inconsistent, and (2) while more studies report changes in walking speeds in stroke patients [35], a substantial heterogeneity exists concerning the time of measurement and inclusion criteria for these studies. As a result, we do not have yet sufficient information to estimate the variance of our principal outcome. Consequently, we plan to assess the variance of the outcomes and the effect sizes with an interim analysis based on 30 subjects. The definitive sample size will be assessed accordingly.

### Statistical analysis

Statistical analyses will be supervised by a skilled statistician and performed with MATLAB and STATA. We will analyze all randomized participants for whom the T1 assessment of the primary outcome and at least one training session are available (i.e., the “intention-to-treat” principle). The interim analysis, including 30 participants, will guide the final statistical methodology. The short-term efficacy of the intervention will be analyzed using the changes between T1 and T0. As the null hypothesis, we postulate that the change before and after the intervention (T1–T0) will not differ between the two groups. We will use simple comparison tests (*t* tests) and multiple regressions; in this case, intervention intensity (number of training sessions), disease etiology, and age will be used as covariates. Furthermore, we will longitudinally analyze (linear growth model) the gait parameters recorded in each gait training session; we hypothesize (H0) that the change over time will be equivalent between groups. We will also assess the difference between groups for the outcomes that are measured at T2 (3-month follow-up). Finally, as a subgroup analysis, we plan to analyze stroke survivors, TBI, and SCI patients separately.

### Safety and confidentiality

Fall risks in frail patients are inherent to walking practice. However, the benefits of high-intensity training far outweigh the consequences of an isolated fall. We take several safety measures to mitigate fall risks during the intervention. The participants are constantly supervised by skilled therapists during the interventions and assessments. The participants can use handrails to stabilize their gait during the treadmill intervention. The intervention always begins with the Zebris treadmill, which is equipped with a safety harness; the participants continue with the C-Mill treadmill only if their gaits are sufficiently stabilized.

Other potential burdens may include physical fatigue and muscle pain because of the repetitive practice of exercises. Although this is an inevitable consequence of gait rehabilitation, we will take into account the complaints of the patients by modulating the intensity and frequency of the training sessions. Finally, it cannot be totally excluded that participants in the AR intervention may experience dizziness or vertigo, especially if they have balance disorders. This effect has never been reported in the literature, but we are ready to document and account for this potential adverse event.

Individual subject medical information obtained as a result of this study is considered confidential, and disclosure to third parties is prohibited. During the trial, confidentiality is ensured by utilizing subject identification code numbers. In the final stage of the study, a full anonymization procedure will be conducted to build the final database. All participants are specifically informed about this procedure.

## Discussion

Particular attention has been paid to the study’s pragmatic design, to ensure that it guarantees high generalizability and yields useful findings. We evaluated protocol design according to the rating grid proposed by the PRagmatic Explanatory Continuum Indicator Summary (PRECIS)-2 guidelines [28] (Table 4). The average score among the nine PRECIS domains is 4.33, which indicates a rather pragmatic protocol. Issues that tend to lower the score are: (1) the focus on a subpopulation of neurologic patients, and (2) the fact that the trial takes place in a single center with some particularities (high-income country, over-representation of traumatic patients).

**Table 4:** PRagmatic Explanatory Continuum Indicator Summary (PRECIS-2) scores of trial domains

	Domain	Score	Rationale
1	Eligibility criteria	3	P: inclusion of patients with gait disorders whatever the etiology
			E: focus on acute to subacute patients in first phase of rehabilitation
2	Recruitment path	4	P: recruitment of participants through usual clinical care at admission
			E: specific procedures to assess eligibility (3 weeks of monitoring)
3	Setting	3	P: study takes place in a neurorehabilitation unit
			E: single-center trial in a high-income country
4	Organization intervention	5	P: resources, provider expertise, and organization of care delivery in both study arms are similar to usual care
5	Flexibility of experimental intervention: delivery	5	P: identical flexibility to usual care
6	Flexibility of experimental intervention: adherence	5	P: no more than usual encouragement to adhere to the intervention
7	Follow-up	4	P: assessment of outcomes through usual clinical care
			E: specific procedure for follow-up after discharge
8	Outcome	5	P: outcomes are of obvious importance to participants and caregivers
9	Analysis	5	P: intention-to-treat analysis with all available data

Score: 1 Very explanatory; 2 Rather explanatory; 3 Equally pragmatic/explanatory; 4 Rather pragmatic; 5 Very pragmatic. *P* pragmatic, *E* explanatory

We hope that the study’s findings will provide knowledge about recovery in neurological patients and help in designing better rehabilitation programs to accompany this process. In particular, the instrumented treadmills will collect a large amount of gait data, which is of interest for further research in neurorehabilitation. Practitioners will benefit from new information for tailoring gait interventions to the needs of their patients. Finally, the results will also help health care funders to decide whether using treadmills equipped with AR capabilities is a worthwhile investment.

### Trial status

The GASPAR trial began recruitment in June 2016.

## Abbreviations

AR: Augmented reality
CRR: Clinique Romande de Réadaptation
GASPAR: Gait Adaptation for Stroke Patients with Augmented Reality
IRR: Institute for Research in Rehabilitation
PRECIS: PRagmatic Explanatory Continuum Indicator Summary
SCI: Spinal cord injury
SPIRIT: Standard Protocol Items: Recommendations for Interventional Trials
TBI: Traumatic brain injury

## References

[1] Meyer K, Simmet A, Arnold M, Mattle H, Nedeltchev K (2009). Stroke events and case fatalities in Switzerland based on hospital statistics and cause of death statistics. Swiss Med Wkly.

[2] Feigin VL, Forouzanfar MH, Krishnamurthi R, Mensah GA, Connor M, Bennett DA (2014). Global and regional burden of stroke during 1990–2010: findings from the Global Burden of Disease Study 2010. Lancet.

[3] Rosamond W, Flegal K, Furie K, Go A, Greenlund K, Haase N (2008). Heart disease and stroke statistics—2008 update. Circulation.

[4] Walder B, Haller G, Rebetez MML, Delhumeau C, Bottequin E, Schoettker P (2013). Severe traumatic brain injury in a high-income country: an epidemiological study. J Neurotrauma.

[5] Chamberlain JD, Deriaz O, Hund-Georgiadis M, Meier S, Scheel-Sailer A, Schubert M (2015). Epidemiology and contemporary risk profile of traumatic spinal cord injury in Switzerland. Inj Epidemiol.

[6] Lam T, Eng JJ, Wolfe DL, Hsieh JT, Whittaker M, Team SR (2007). A systematic review of the efficacy of gait rehabilitation strategies for spinal cord injury. Top Spinal Cord Inj Rehabil.

[7] Jørgensen H, Nakayama H, Raaschou H, Olsen T (1999). Stroke. Neurologic and functional recovery the Copenhagen Stroke Study. Phys Med Rehabil Clin N Am.

[8] van den Brand R, Heutschi J, Barraud Q, DiGiovanna J, Bartholdi K, Huerlimann M (2012). Restoring voluntary control of locomotion after paralyzing spinal cord injury. Science.

[9] Murphy TH, Corbett D (2009). Plasticity during stroke recovery: from synapse to behaviour. Nat Rev Neurosci.

[10] Krakauer JW, Carmichael ST, Corbett D, Wittenberg GF (2012). Getting neurorehabilitation right what can be learned from animal models?. Neurorehabil Neural Repair.

[11] Langhorne P, Bernhardt J, Kwakkel G (2011). Stroke rehabilitation. Lancet.

[12] Breceda EY, Dromerick AW (2013). Motor rehabilitation in stroke and traumatic brain injury: stimulating and intense. Curr Opin Neurol.

[13] Langhorne P, Coupar F, Pollock A (2009). Motor recovery after stroke: a systematic review. Lancet Neurol.

[14] Polese JC, Ada L, Dean CM, Nascimento LR, Teixeira-Salmela LF (2013). Treadmill training is effective for ambulatory adults with stroke: a systematic review. J Physiother.

[15] Peurala SH, Karttunen AH, Sjögren T, Paltamaa J, Heinonen A (2014). Evidence for the effectiveness of walking training on walking and self-care after stroke: a systematic review and meta-analysis of randomized controlled trials. J Rehabil Med.

[16] Dickstein R (2008). Rehabilitation of gait speed after stroke: a critical review of intervention approaches. Neurorehabil Neural Repair.

[17] van Hedel HJ, Dietz V (2010). Rehabilitation of locomotion after spinal cord injury. Restor Neurol Neurosci.

[18] Nyberg L, Gustafson Y (1995). Patient falls in stroke rehabilitation. A challenge to rehabilitation strategies. Stroke.

[19] McCulloch KL, Buxton E, Hackney J, Lowers S (2010). Balance, attention, and dual‐task performance during walking after brain injury: associations with falls history. J Head Trauma Rehabil.

[20] Brotherton SS, Krause JS, Nietert PJ (2007). Falls in individuals with incomplete spinal cord injury. Spinal Cord.

[21] Kenzie JM, Semrau JA, Findlater SE, Herter TM, Hill MD, Scott SH (2014). Anatomical correlates of proprioceptive impairments following acute stroke: a case series. J Neurol Sci.

[22] Lawrence ES, Coshall C, Dundas R, Stewart J, Rudd AG, Howard R (2001). Estimates of the prevalence of acute stroke impairments and disability in a multiethnic population. Stroke.

[23] Smania N, Picelli A, Gandolfi M, Fiaschi A, Tinazzi M (2008). Rehabilitation of sensorimotor integration deficits in balance impairment of patients with stroke hemiparesis: a before/after pilot study. Neurol Sci.

[24] Patterson KK, Mansfield A, Biasin L, Brunton K, Inness EL, McIlroy WE (2014). Longitudinal changes in poststroke spatiotemporal gait asymmetry over inpatient rehabilitation. Neurorehabil Neural Repair.

[25] Hollands KL, Pelton TA, Tyson SF, Hollands MA, van Vliet PM (2012). Interventions for coordination of walking following stroke: systematic review. Gait Posture.

[26] Nascimento LR, de Oliveira CQ, Ada L, Michaelsen SM, Teixeira-Salmela LF (2015). Walking training with cueing of cadence improves walking speed and stride length after stroke more than walking training alone: a systematic review. J Physiother.

[27] Heeren A, van Ooijen MW, Geurts AC, Day BL, Janssen TW, Beek PJ (2013). Step by step: a proof of concept study of C-Mill gait adaptability training in the chronic phase after stroke. J Rehabil Med.

[28] Loudon K, Treweek S, Sullivan F, Donnan P, Thorpe KE, Zwarenstein M (2015). The PRECIS-2 tool: designing trials that are fit for purpose. BMJ.

[29] Chan A-W, Tetzlaff JM, Altman DG, Laupacis A, Gøtzsche PC, Krleža-Jerić K (2013). SPIRIT 2013 statement: defining standard protocol items for clinical trials. Ann Intern Med.

[30] RALLOC: Stata module to design randomized controlled trials. http://EconPapers.repec.org/RePEc:boc:bocode:s319901. Accessed 10 Jun 2016.

[31] Mills EJ, Seely D, Rachlis B, Griffith L, Wu P, Wilson K (2006). Barriers to participation in clinical trials of cancer: a meta-analysis and systematic review of patient-reported factors. Lancet Oncol.

[32] Schlick C, Ernst A, Bötzel K, Plate A, Pelykh O, Ilmberger J (2016). Visual cues combined with treadmill training to improve gait performance in Parkinson’s disease: a pilot randomized controlled trial. Clin Rehabil.

[33] van Ooijen MW, Heeren A, Smulders K, Geurts AC, Janssen TW, Beek PJ (2015). Improved gait adjustments after gait adaptability training are associated with reduced attentional demands in persons with stroke. Exp Brain Res.

[34] Hollands KL, Pelton TA, Wimperis A, Whitham D, Tan W, Jowett S (2015). Feasibility and preliminary efficacy of visual cue training to improve adaptability of walking after stroke: multi-centre, single-blind randomised control pilot trial. PLoS One.

[35] Swiss Heart Foundation. http://www.swissheart.ch/. Accessed 10 Jun 2016.

[36] Pin TW (2014). Psychometric properties of 2-minute walk test: a systematic review. Arch Phys Med Rehabil.

[37] Blum L, Korner-Bitensky N (2008). Usefulness of the Berg Balance Scale in stroke rehabilitation: a systematic review. Phys Ther.

[38] Büla CJ, Martin E, Rochat S, Piot-Ziegler C (2008). Validation of an adapted Falls Efficacy Scale in older rehabilitation patients. Arch Phys Med Rehabil.

[39] Ware JE, Gandek B (1998). Overview of the SF-36 health survey and the International Quality of Life Assessment (IQOLA) project. J Clin Epidemiol.

